# The course of depressive symptoms during the postmenopause: a review

**DOI:** 10.1186/s40695-015-0003-x

**Published:** 2015-08-11

**Authors:** Katherine E Campbell, Cassandra E. Szoeke, Lorraine Dennerstein

**Affiliations:** 1 Department of Psychiatry, University of Melbourne, Victoria, Australia; 2 Department of Medicine, University of Melbourne, Victoria, Australia

**Keywords:** Postmenopause, Depressive symptoms, Centre for Epidemiological Studies Depression Scale (CESD)

## Abstract

As the Australian population ages, significantly more women are entering the postmenopausal stage of the climacteric, yet research focusing on the prevalence of depressive symptoms in this stage of ovarian ageing is scarce. This review will examine the information provided by studies that have a cohort with data of adequate duration to explore depressive symptom prevalence in the early and late postmenopause. Longitudinal epidemiological studies of women transitioning through the postmenopause that included measures of mood and/or depressive symptoms were identified through searches of MEDLINE (1980-2014) and PsycINFO (1980-2014) databases. Population based studies with at least two time points of assessment were included. Longitudinal studies of ageing that did not categorise women as postmenopausal were not included, as this was outside the scope of this review.

Prevalence estimates of depressive symptoms varied between studies and ranged from 8.5 % to 25.7 % with percentages between 22 and 25 % being most consistently reported. Surgical postmenopause groups reported higher ratings of depressive symptoms at 18-42 % and higher incidence of major depressive disorder in all but one study. The prevalence of Major Depressive Disorder also varied with ranges from <1 % to 42 % reported. Wide ranges in prevalence were reported in the literature. Differences in definitions, inconsistent sample sizes and varying measures make it difficult to compare results across studies. The specific inclusions and exclusions of sub-samples of larger cohorts are at times inconsistent with epidemiological acquisition and, as such, impact upon generalizability of results to a healthy population.

## Background

Research consistently demonstrates a higher occurrence of depressive disorders and depressive symptoms in women compared to men [1, 2]. This difference has been demonstrated in a variety of contexts including population studies, hospital admissions, suicide attempts and the prescription of anti-depressant medication [3]. The gender difference begins during adolescence and continues into old age, corresponding roughly with a woman’s reproductive years [4]. It has been proposed that changes in ovarian sex steroids may be a contributing factor in the higher vulnerability for women to develop a depressive disorder [5]. For this reason it has been suggested that there are certain windows of vulnerability for the development of depression across the lifespan and there has been significant research conducted into the characteristics of depression in women in specific age ranges or during biological transitions such as adolescence, the postpartum period and late-life [6].

There are a number of studies which have examined mood in the menopausal transition, a time representing significant changes in physiology associated with ovarian ageing [7, 8]. However, there is limited literature examining the period of time directly following the final menstrual period, a physiological marker corresponding to the onset of early postmenopause. With the recent updates to the staging system used to characterise menopausal status [9], the consistent definition of the postmenopausal stages will allow for a greater understanding of depressive symptoms in this particular stage of reproductive ageing.

Unique hormonal changes take place in the first two years following the final menstrual period and coincide with the early postmenopausal stage of reproductive ageing as outlined in the new staging system used to characterise the menopausal transition [9]. While the final menstrual period (FMP) is a distinct and measurable physiological event in the reproductive aging cycle, it is not commonly used as a reference point for understanding the temporal characteristics of depressive symptoms across the postmenopausal period. Given the variability of length of certain menopausal stages, the FMP could serve as a consistent marker for future research examining mood in the menopause. If uniformly included in future publications, calculating years preceding or following FMP would allow for comparability across studies regardless of the definition used to categorize the menopausal stage. As our understanding of ovarian ageing advances, this physiological marker would remain a stable frame of reference regardless of amendments to the broader reproductive ageing stages.

Several longitudinal epidemiological studies were initiated to specifically examine the association between mood and menopause [10–12]. As these studies have matured those remaining now have data available on women who have entered the postmenopausal period and in some cases transitioned from early postmenopause into late postmenopause [12, 13]. The data provided in these longitudinal studies will improve our understanding of the characteristics of depressive symptoms in the postmenopausal period.

It has been estimated that by 2030, 1.2 billion women will be postmenopausal, compared to 470 million in 1990 [14]. The onset of postmenopause is variable due to the individual discrepancy in the occurrence of the final menstrual period. As the average age of postmenopause onset is between 50 and 52 years and the current life expectancy of a female in high-income countries is 82 years [15], the length of postmenopause is, on average, approximately thirty years in developed countries. As the female population ages, an increasing number of women will be experiencing postmenopause and an understanding of the risk factors associated with this period will become increasingly important.

The postmenopausal period can encompass up to a third of a woman’s life, yet the distinction between the early and late stages of the postmenopause are rarely focused upon. Recent research examining the ongoing changes in estradiol and follicle stimulating hormone (FSH) levels demonstrate that distinct and observable changes in hormone levels continue for several years after the perimenopause and into the postmenopause [16]. Consistent endocrinological patterns were used to determine the reproductive stages of ageing staging system [9]. Randolph and colleagues demonstrated that the most rapid changes in FSH and estradiol occur in the two years preceding and two years following the final menstrual period, regardless of the age at which FMP occurs [17]. This finding highlights the need to distinguish between ovarian ageing and chronological ageing when exploring the impact of menopausal status on depressive symptoms. The experience of the final menstrual period is an essential reference point in determining if changes in sexual hormones affect the risk of experiencing depressive symptoms independent of age. The recent changes in the understanding of the early postmenopause, and in turn the classification system used to describe it, emphasises the need for further study of this specific stage of the menopausal transition. Despite this, few studies identify the early postmenopause substages as periods of ovarian ageing with distinct physiological profiles. Rather the early postmenopause is often defined with arbitrary time frames or not defined at all [18]. Research examining depressive symptoms and mood across the early postmenopause, using the updated criteria, is needed.

Reproductive endocrine function has stabilised by late postmenopause [9]. Few studies of the menopausal transition have continued to follow women into late postmenopause. Women experiencing the postmenopause are entering late-life and research on depressive symptoms in this population focuses on somatic concerns and other physiological factors associated with ageing [19]. As data from longitudinal studies becomes available the impact of ovarian ageing on mood from midlife to late-life can be studied specifically.

Midlife transitions can have both a positive and negative impact on mental health issues [20]. Caregiving, changes in marital status and perceived health decline are examples of life transitions that have been shown to increase depressive symptom development in midlife women [20]. Similarly, changes in lifestyle factors and social circumstances that occur between the early stages and late stages of postmenopause may also impact the development of depressive symptoms.

### Postmenopause classification

**Fig. 1:**
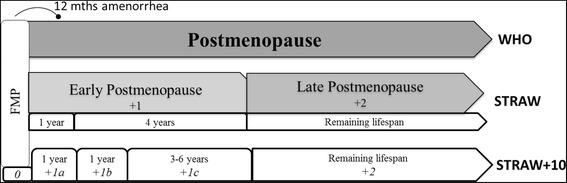
Stages of postmenopause with corresponding WHO, STRAW and STRAW + 10 criteria as described in text

The most commonly used menopausal status classification systems are the World Health Organisation (WHO) criteria [21] and that developed at the original Stages of Ageing Reproductive Workshop (STRAW) [22]. Both systems define ‘postmenopause’ as beginning after the final menstrual period (FMP) and continuing for the remainder of a woman’s life [9]. The STRAW criteria, and the updated 2011 criteria (STRAW + 10), offer a further breakdown of the postmenopausal transition due to the length of the postmenopausal period. The categories outlined in the STRAW + 10 staging system are illustrated in Fig. 1 [9]. Despite this distinction few epidemiological studies distinguish between early and late postmenopause when reporting on prevalence of depressive symptoms during this period [18, 23, 24].

The early postmenopause stage has been organised into three substages: the first two of one year duration and the third stage varying between three and six years. The first stage is determined retrospectively and represents the twelve months of amenorrhea following the final menstrual period (FMP). Early postmenopause includes a period in which the FSH levels increase and estradiol levels rapidly decrease and move toward stabilisation. Vasomotor symptoms are most likely to occur during this time due to the radical fluctuation in hormones [9].

The third substage of the early postmenopause represents the period of stabilisation of low levels of estradiol and high levels of FSH. Further research is needed to determine the average timing and specific trajectories of change at which stabilisation occurs, however it is thought to be between three and six years. Due to the varied length of the third substage, the overall length of early postmenopause ranges between five and eight years [9].

Late postmenopause follows the early postmenopause stage and continues for the remainder of a woman’s life. In this stage reproductive endocrine function has stabilised and further changes in sex steroids are limited [9]. It has been suggested that further research may be required to determine if further declines in FSH occur in very old women [9].

### Defining the postmenopause in examining depression

Despite the clear distinction in physiological profile between the early postmenopausal substages and late postmenopause the majority of studies examining the occurrence of depressive symptoms during postmenopause have not distinguished between these periods and include only a general distinction between early and late postmenopause [13] or no distinction at all [18, 25].

In order to better understand the trajectories of depressive symptom ratings across the postmenopause each of the unique substages of the early and late postmenopause need to be considered. Using the general term ‘postmenopause’ to compare longitudinal data will not adequately capture the potential risks associated with the distinct substages of the transition.

Similarly a clear distinction needs to be made between women who experience surgical menopause and those that have a natural menopause [10, 25, 26]. Surgically postmenopausal women have been shown to demonstrate higher scores on measures of depressive symptomology [10, 25, 26]. For this reason any reports of prevalence of depressive symptoms in postmenopausal women needs to consider women who entered the postmenopause due to oophorectomy or hysterectomy as a separate group.

The goal of the following review is to examine the current information available regarding the occurrence of depressive symptoms across the postmenopausal period and to determine research questions where gaps exist in the literature.

## Methods

PsychINFO (1970-2014) and MEDLINE (1975-2014) databases were searched using the following key terms: ‘depression’, ‘depressive disorder’, ‘depressive symptom’, ‘negative mood’, ‘negative affect’, ‘postmenopause’, ‘postmenopausal’, ‘menopausal transition’ and ‘menopause’. Search results of 648 (PsycINFO) and 9,780 (MEDLINE) articles were initially identified through searches using various combinations of the key terms. These were limited to articles published in English and screened for studies describing longitudinal epidemiological studies of women that had data of adequate duration to report on prevalence of depressive symptoms or depressive disorder during the postmenopause. Studies that included information on risk of experiencing depressive symptoms or depression during postmenopause were also included. Studies exploring major depressive disorder, major depressive episode, mood disorders and depressive symptoms were all reviewed to capture any potential studies for inclusion. Broad search terms were used due to the inconsistency in the definitions and measures being used between, and even within the longitudinal studies. A manual search of journal articles and bibliographies was also conducted to ensure that all relevant materials were examined. This search yielded an additional 200 articles. All abstracts were reviewed for content relevant to prevalence of depressive symptoms in the postmenopausal period.

Longitudinal population based studies of mood and menopause were included if they had at least two time points of assessment with the same cohort, if they specifically defined a postmenopausal group independent of other menopausal status groups, and if they included a standardised measure of self-reported depressive symptoms or a clinically recognised diagnostic tool for assessing Major Depressive Disorder. The following studies were included: the Women’s Healthy Ageing Study (WHAP); the Seattle Midlife Women’s Health Study (SWMHS); the Study of Women’s Health Across the Nation (SWAN); the Manitoba Project; the Penn Ovarian Ageing Study (POAS); and the Eindhoven Perimenopausal Osteoporosis Study (EPOS). Only articles with data pertaining specifically to prevalence or standardised reports of increased risk of depressive symptoms or depressive disorder in the postmenopause are described in this review. Inconsistency in methodology, sample sizes, and analysis techniques made it unreasonable to conduct a meta-analysis without excluding important studies reporting on prevalence.

A number of different measures of depression and depressive symptoms were used by the different longitudinal studies. The most consistently used measure to assess depressive symptom severity was the Centre for Epidemiological Studies Depression Scale (CESD). The CES-D is a self-report scale specifically designed to be used in epidemiological studies to assess the presence of clinical and non-clinical symptoms of depression in the general population. It is one of the most commonly used depression measure in studies of healthy populations [27]. Items assess four areas related to depression: depressed affect, positive affect, somatic activity and interpersonal aspects [28]. The cut off ranges used to categorise normal versus mild to moderate symptoms of depression for the short and full versions are ≥10 and ≥16 respectively [27, 29]. The only other depressive symptom measure used was the Edinburgh Depression Scale, originally developed for use with post-natal women but validated for use with menopausal women [30]. Cut offs of 12 or more are used to determine risk of depression [18].

The Structured Clinical Interview for DSM-IV Axis 1 Disorders (SCID) [31], and Primary Care Evaluation of Mental Disorders (PRIME-MD) [32] were used to assess presence of depressive disorders. The SCID is a clinician administered diagnostic tool used to assess for the presence of past or current major depressive disorders based on DSM criteria. The PRIME-MD is a standardised, validated diagnostic assessment used to diagnose mood, anxiety, alcohol and eating disorders, also using DSM criteria.

## Results

A summary of sample sizes, participant exclusion criteria, definition of postmenopausal stage and the measurements used varied between, and at times within, the larger cohort studies and have been outlined in Table 1.

**Table 1:** Longitudinal cohort summary of depressive symptom incidence

Cohort Details		SubSample characteristics	Follow-Up Type and Duration	Sample and Assessment of Depression	Definition of Postmenopause/ PoM Range	Results
Penn Ovarian Ageing Study (POAS)**—**Baseline cross section 436 women 35-47 yrs, from Pennsylvania USA, recruited 1996**.**	Freeman et al., 2004 [33].	PoM *n* = 11	Six assessments at eight month intervals over four years.	Depressive symptoms:	STRAW PoM Maximum Duration: 11 years	CESD mean score 10.6 (pre 12.7, early transition 14.6, late transition 13.1)
				CESD < 16 vs ≥16		
				Depressive Disorder: Primary Care Evaluation of Mental Disorders (PRIME-MD).		
						Diagnosis of MDD <1 %
	Freeman et al., 2014 [34].	PoM *n* = 203	Longitudinal; 14 year FU	Depressive symptoms: CESD < 16; ≥16;≥25.	STRAW + 10; PoM Maximum Duration: 14 years	CESD of 16 or greater decreased approximately 15 % each year following FMP.
				Depressive Disorder: Primary Care Evaluation of Mental Disorders (PRIME-MD).		
					Also included analysis using years since FMP as reference.	
						OR for risk of depressive symptoms highest for first two years following FMP.
						~35 % of women with a history of depression reported high scores in each postmenopausal year compared to 0-15 % of women with no history.
Study of Women’s Health Across the Nation (SWAN)**—**Baseline cross section 16,065 women 40-55 yrs, from 7 geographic	Bromberger, et al., 2007 [35].	*n* = 2885 (25 % PoM)	Longitudinal; 5 yr FU	Depressive symptoms: CESD < 16 vs ≥16	WHO PoM Maximum Duration: 6 years	OR of having CESD score ≥16 in postmenopause (1.57) → significantly higher than premenopause.
		PoM *n* = ~721				
regions across the USA, recruited 1995-1997.	Bromberger et al., 2010 [11].	*n* = 3296 (66 % PoM) PoM *n* = ~2175	Longitudinal; 8 yr FU	Depressive symptoms: CESD < 16 vs ≥16	WHO; STRAW PoM Maximum Duration: 9 years	OR of having CESD score ≥16 in postmenopause (1.79) → significantly higher than premenopause.
	Bromberger et al., 2011 [13].	*n* = 221 Ancillary SWAN study – Mental Health Study (MHS) – Pittsburgh site. PoM *n* = 131	Longitudinal; 10 yr FU	Major Depressive Episode: SCID	Early PoM: (≤2 yrs amenorrheic)	PoM 9.8 %.
						Early PoM significantly greater risk for MDE than premenopause
					Late PoM: (≥2 yrs amenorrheic)	
					PoM Maximum Duration: 11 years	
	Joffe et al., 2012 [26].	*n* = 425 Ancillary SWAN study (MHS) Pittsburgh site.	Longitudinal; 6 yr FU	Past or current Depressive Disorder: SCID	WHO PoM Maximum Duration: 7 years	PoM: 64/151 (42.4 %) met criteria
						Surgical PoM: 12/38 (31.6 %) met criteria
		PoM *n* = 151 Surgical PoM n = 38				
Melbourne Women’s Midlife Health Project (MWMHP)/Women’s Healthy Ageing Study (WHAP) Baseline cross-section 2001	Dennerstein et al., 2004 [10].	314 women PoM = 207 Surgical PoM = 39	Longitudinal; initial year of CESD assessment 1991. FU 11 years.	Depressive symptoms: CESD(Brief) < 10 vs ≥10	STRAW PoM Maximum Duration: 11 years	CESD ≥ 10
						PoM: 22 %
						Surgical PoM: 42 %
						Mean CESD Score:
						PoM: 6.7
						Surgical PoM: 8.7
women aged 45-55 yrs, from Melbourne Australia, recruited 1990-1991.	Ryan et al., 2009 [5].	PoM = 138	Longitudinal; 2 yr FU	Depressive symptoms: CESD(Brief) < 10 vs ≥10	STRAW PoM Maximum Duration: 13 years	CESD ≥ 10
						PoM: 25.4 %
			2002 compared to 2004.			
Seattle Midlife Women’s Health Study (SMWHS) Baseline cross section 508 (35-55 yrs mean age 41 yrs), from Seattle USA, recruited 1990-1992.	Woods et al., 2008 [39].	PoM *n* = 87	Baseline and 15 year FU.	Depressive symptoms: CESD < 16 vs ≥16	STRAW Early PoM: 5 yrs from FMP	No significant difference in CESD score between menopausal stages.
					PoM Maximum Duration: 5 years	
						Small but significant decrease of 0.10 in CESD score each year.
Eindhoven Perimenopausal Osteoporosis Study (EPOS) Baseline recruitment 1994 -1995. Women born between 1941 and 1947 from Eindhoven, Netherlands.	Maartens et al., 2002 [18].	T1: 1995 PoM: *n* = 646	Assessment in 1995 and 1998, approximately 3.5 years apart.	Depressive symptoms: Edinburgh Depression Scale (EDS) < 12 vs ≥12	Amenorrhea for at least one year.	T1 N = 646 - Mean EDS: 7.8
		T2: 1998 PoM *n* = 1379			PoM Maximum Duration: unknown	EDS > 12 -- > 24.2 %
						T2 N = 1379 - Mean EDS 7.7 EDS >12 -- > 25.7 %
The Manitoba Project— Baseline cross section 2500 (40-59 yrs),from Manitoba Canada, recruited 1982-1985.	Kaufert, Gilbert & Hassard, 1988 [41].	T1-T6 PoM;.	Baseline and 3 years FU with 6 monthly contact. 6 time points.	Depressive symptoms: CESD < 16 vs ≥16	12 months without menstruation.	No significant difference in CESD score between menopausal stages.
		*n* = 5, 5, 18, 24, 29, 35				
					PoM Maximum Duration: 3 years	
	Kaufert, Gilbert & Tate, 1992 [42].	T1-T5 PoM.	First five time points.	Depressive symptoms: CESD < 16 vs ≥16	12 months without menstruation.	Relative odds of depression (compared to pre and peri):
		*n* = 5, 5, 18, 24, 29				
					PoM Maximum Duration: 2.5 years	
						Non-depressed PoM: 0.87
						Depressive history PoM: 0.84
						Hysterectomised non-depressed PoM: 1.7
						Hysterectomised depressive history PoM: 0.84

Prevalence ratings of depressive symptoms or major depressive disorder by cohort. Original cohort baseline details provided furthest left. Details of subsample of cohort used for each article reported separately for purposes of clarity

PoM – postmenopause; ~ - approximately; SurPoM – surgical postmenopause; FU – Follow-up; T - Time point; CESD – Centre for Epidemiological Studies Depression Scale; SCID - Structured Clinical Interview for DSM Disorders; OR – odds ratio

### The Penn Ovarian Ageing Study

The Penn Ovarian Ageing Study (POAS) began recruitment in 1996. A baseline cross sectional sample of 436 women aged between 35 and 47 years from Pennsylvania were recruited [33]. The findings from the Penn study include results from the assessment of both depressive symptom scores and clinical diagnosis of Major Depressive Disorder. Symptoms scores were based on the CESD with cut off of at or above 16. Clinical diagnosis was made using the Primary Care Evaluation of Mental Disorders (PRIME-MD) assessment tool which determined a diagnosis of current mood disorder at each period of assessment. Six assessments were conducted at eight month intervals across approximately four years. As postmenopausal women were excluded at baseline, the maximum length of time since final menstrual period was approximately four years. The age of the postmenopausal group ranged from 44 to 51 years. The mean CESD scores for the postmenopausal group as they transitioned through the third, fourth and fifth assessment periods were: 1.0 (*n* = 2); 6.0 (*n* = 4); and 13.8 (*n* = 9). Participants did not meet criteria for Major Depressive Disorder based on the PRIME-MD at any of these time points. The mean CESD score for the group at the sixth assessment period was 10.6 (normal range) and less than 1 % met criteria for diagnosis of Major Depressive Disorder. Participants were considered as having a history of depression if they met criteria for MDD based on the PRIME-MD at any assessment point in the study. Results of the 2004 study need to be interpreted with caution due to the small sample size, with only 11 women meeting STRAW criteria for postmenopause classification [33].

A much larger sample size was included in the 2014 POAS study, with 203 postmenopausal women included in the analysis [34]. The sample was divided into groups, with CESD scores being reported separately for women with a history of depression (*n* = 90) and women with no history (*n* = 113). History of depression was determined at baseline based on self reported medical history or as determined by the PRIME-MD. The mean age at baseline was 42.8 years. The mean age at FMP was 51.1 years and ranged from 42-58 years. In this study, time since the final menstrual period was used as a marker to determine changing symptom prevalence scores across the years of the postmenopause, with data reported annually up to 7 years post FMP then grouped as ‘greater than 8 years’. Overall the number of scores on the CESD of 16 or greater (high scores) decreased approximately 15 % each year, suggesting a gradual decline of depressive symptoms. Approximately 35 % of women with a history of depression reported high scores in each postmenopausal year compared to 0-15 % of women with no history. Women with a history of depression had more than 13 times the risk of experiencing depressive symptoms compared to women with no history.

The odds ratio for risk of depressive symptoms following FMP were highest for the first two stages of the early postmenopause, and decreased steadily across the third substage of early postmenopause for women with a history of depression. Participants in the late postmenopausal stage (grouped as ≥8 years since FMP), were more likely to report depressive symptoms than women in the third sub-stage, but not the first two substages, of early postmenopause. For women with no history of depression 1030 % reported a higher CESD before the FMP and experienced a significant decrease after the second year following menopause with prevalence of 0 % to 15 % reported. The 2014 Penn study is the first to use STRAW + 10 criteria and makes use of the final menstrual period as a means of tracking patterns of individual annual scores across the postmenopause.

### The Study of Women’s Health Across the Nation

Of all the longitudinal studies reporting depressive symptom data the SWAN study has the most robust sample size and has distinguished between early and late postmenopause in at least one of their studies [35]. In the SWAN cohort women were recruited between 1995 and 1997 across seven geographic regions of the United States. The SWAN study also includes large samples from multiple ethnicities including Caucasians, African Americans and Americans with Chinese, Japanese and Hispanic backgrounds.

In the earliest SWAN study reporting depressive symptom prevalence, Bromberger and colleagues used CESD cutoffs of <16 and ≥16 and found that of the 721 postmenopausal women the odds ratio of having mild or moderate levels of depressive symptoms was significantly higher in postmenopause compared to premenopause [35]. In this publication, there was no distinction made between early and late postmenopause. The definition of postmenopause was in keeping with the WHO criteria (no menses in the past 12 months) [35]. The baseline age for the entire cohort was 46.5 years (SD 2.7 years) and included an age range between 42 and 52 years. The mean age for the postmenopause group was not provided separately. Eligibility into the study required no surgical menopause and menses within the last three months. As the study included a baseline visit and five subsequent annual visits the maximum length of postmenopause was six years.

Higher odds ratio for postmenopause (1.79) compared to premenopause was demonstrated again in their follow up study where this result was confirmed with a larger sample size of 2175 postmenopausal women [36]. The maximum postmenopausal range increased to nine years in this study which reported on the 8th year of follow-up. In 2011, Bromberger and colleagues presented findings from a 10-year follow-up of a subsample of their larger SWAN cohort and specifically examined whether risk factors and prevalence of Major Depressive Disorder were different during the postmenopausal phase. Of the 221 women in this analysis (all of whom were premenopausal at study entry), 131 had experienced at least one interview in which they were postmenopausal based on WHO criteria [13]. Those women classified as postmenopausal were further categorised as early (≤2 years amenorrheic) or late postmenopausal (>2 years amenorrheic) [30]. Based on the new STRAW + 10 criteria many of these ‘late postmenopause’ participants would remain in the early postmenopause category which lasts up to 8 years since onset of amenorrhea [9]. The average age at baseline, for the women with no depressive history (*n* = 152), was 45.7 years, and with depressive history (*n* = 69) was 45.1 years. The maximum duration of postmenopause observed during the study was eleven years. They found that women who were in the early postmenopausal phase were at greater risk of experiencing a major depressive episode compared to when the women were premenopausal. There was no significant difference for women in the late postmenopausal stage compared to when the women were premenopausal. These findings were based on SCID assessments identifying current or recent major depressive episode measured annually over a ten year period using data from an ancillary SWAN study called the Mental Health Study in which a subset of 221 participants from the Pittsburgh site were included in the analysis [13].

In a more recent study, the SWAN team looked at the relationship between history of depression and quality of life, again using data from the Pittsburgh cohort of the Mental Health Study [26]. In this study 425 women from the Pittsburgh site underwent annual SCID assessments. The data provided lifetime prevalence of depressive disorder as well as menopausal status. The mean age at study entry was 46 years for the entire cohort. The mean age of the postmenopausal women was not provided. Of the 425 women, 151 (35 %) were categorised as naturally postmenopausal (as per WHO categorisations). Of these women 64 (42.4 %) met criteria for having ever had depressive disorder. This high number may reflect the fact that the definition of depressive disorder was broader than with other studies that considered only a diagnosis of Major Depressive Disorder. The SWAN project defined history of depression as including “an episode of major or minor depression that occurred before the current visit and resolved in the past month” and, current depression defined as: “major or minor depressive episode occurring within the past month, were in partial remission of a major depressive episode that began earlier,…. or had a current episode of dysthymia” [26].

While depressive symptom scales are able to provide a spectrum of scores from normal to severe, a diagnostic classification, such as that used in this SWAN analysis, separates participants into either having a lifetime occurrence of depressive disorder or not. The broad definition of what constituted depressive history must be taken into consideration when looking at the prevalence of depressive disorder in postmenopausal women in this cohort.

The use of specific subsamples and changing definitions for diagnostic classification used by SWAN, highlights the need for caution when generalising across studies, or as seen in this case even when generalising within large epidemiological studies that report findings using different subsamples of the original cohort.

### The Women’s Healthy Ageing Project

The Women’s Healthy Ageing Project, initiated as the Melbourne Women’s Midlife Health Project, is a longitudinal prospective epidemiological study that draws upon a cohort of over 400 Melbourne women who were identified through random digit dialling and contacted by phone in 1991 and commenced involvement in a longitudinal study. Assessments were conducted annually between 1991 and 1999 and were readministered in 2002, 2004 and in 2012. The Affectometer, a measure of negative mood was administered at each time point with a specific measure of depressive symptoms introduced in 2002, and readministered in 2004 and 2012. WHAP is one of the longer prospective studies on mood in women and has consistently demonstrated that negative or depressed moods, as measured by the Affectometer and the CESD, decline as women age and become postmenopausal [37].

In the WHAP cohort depressive symptoms were assessed in 2002, 2004 and 2012 with the Centre for Epidemiological Studies Depression Scale (CESD), 10 item version [38]. A cut-off of 10 was used to distinguish presence of mild or moderate depressive symptoms from normal levels. The criteria used to determine postmenopausal status were based on the STRAW criteria [22]. In 2002, in the 11th year of follow-up, a total of 314 women completed the CESD 10 items. The mean age of the overall cohort was 59.9 years (range 56-67years). Of the 314 women 207 of the cohort had experienced natural menopause and 39 had undergone surgical menopause. CESD scores indicating mild or moderate symptoms of depression were present in 22 % for the postmenopausal women and 42 % of the surgical postmenopause group with mean scores of 6.7 and 8.7 respectively [10].

In 2004, a subsample of 138 postmenopausal women were included in an analysis of endogenous hormones and depressive symptoms [5]. Surgically postmenopausal women were excluded from analysis. The mean age for the women at the 11th year of follow-up was 60.1 years, ranging from 55.9 to 66.8 years. In the 13th year of follow up, the mean age for the cohort with CESD scores less than 10 (*n* = 103) was 60.3 years and mean years since FMP was 7.0 (SD, 2.3). For those with scores greater than 10 (*n* = 35) mean age was 59.2 with mean years since FMP being 5.9 years (SD 2.5). Of the total postmenopausal sample 25.4 % scored at or above 10 on the CESD [5]. In this sample increased risk of depression symptoms was associated with a decline in total serum estradiol (OR: 3.5; 95 % CI: 1.2-9.9) and with a large increase in FSH levels (OR: 2.6; 95 % CI: 1.0-6.7).

### The Seattle Midlife Women’s Health Study

The Seattle Midlife Women’s Health Study initially recruited 508 participants between 1990 and 1992 [39]. A subset of 390 women from the original cohort continued to provide data across a 15 year period. In 2008, Woods and colleagues summarised the findings exploring depressed mood across the menopausal transition and into early postmenopause [36]. The overall sample size described in this article was 302, with 87 women assessed as being in early postmenopause, defined by the original STRAW criteria as the first five years since FMP.

This study was the only one to report findings for early postmenopause specifically using the original STRAW criteria. The age range at baseline was 35–55 years with a mean age of 41.4 years. The mean age score for the early postmenopause group was not provided. Woods and colleagues found that age was modestly and negatively related to depressed mood with scores of CES-D decreasing with each year of age. While age was a significant predictor of CES-D score and menopausal stage was not, in models that included stress and family history of depression, age ceased to be a significant predictor. The data indicated a rise in depressive symptom scores in the late menopausal transition stage with a slight trend toward lower CESD scores in the early postmenopause compared with the late reproductive stage. The early postmenopausal stage was not found to be significantly different from other stages of the menopausal transition in relation to higher CESD score.

### The Eindhoven Perimenopausal Osteoporosis Study

The Eindhoven Perimenopausal Osteoporosis Study (EPOS) originally recruited 6648 women born between 1941 and 1947 living in Eindhoven in the Netherlands. The longitudinal phase of the study included 2748 women from this cohort. The baseline assessment was conducted in 1995 with follow-up taking place in 1998, approximately 3.5 years after the initial assessment [18]. In the EPOS study depressive symptomatology was assessed using the Edinburgh Depression Scale (EDS) which commonly uses a cut-off of 12 to identify those at risk for depression [40]. Women were categorised into menopausal status with postmenopause women being those who had amenorrhea for at least a year. Women using hormone therapy (HT) or who had undergone surgical menopause were excluded from analysis. At the first time point of assessment in 1995, 646 postmenopausal women with an average age of 51.3 years completed the EDS. The mean score was 7.8 (SD 6.3) and 24.2 % scored at or above 12 [18].

At the second assessment carried out in 1998, 1379 women were postmenopausal with a mean age of 55.5 years. The mean score on the EDS was 7.7 (SD 6.3) and 25.7 % scores were 12 or above. While the mean score for the EDS was slightly lower at the second time point a higher percentage of women scored 12 or above. An estimation of the range of years since final menstrual period could not be determined as postmenopausal women were included at baseline and no reference was made as to how long they had been postmenopausal.

### The Manitoba Project

The Manitoba Project on Women and Their Health initially began as a cross-sectional mail survey of 2500 women aged 40-59 living in Manitoba, Canada [41]. Of the original cross-sectional cohort, 505 women were asked and agreed to continue in the longitudinal phase of the study. The cohort was assessed via phone interview at six time points spaced six months apart over three years between 1982 and 1985. The CESD was used to determine depressive symptom level and postmenopause was defined as 12 months or more without menstruation.

In analysing 330 women in the cohort who were not hysterectomised at baseline and who had completed CESD at all time-points, no association was shown between higher CESD scores and postmenopause status [41]. As would be expected the sample size for postmenopausal women varied over the study, ranging from 17 (5 %) at the first time point to 116 (35 %) at the sixth time point. When examining the entire cohort (pre, peri and postmenopausal women) the percentage of women scoring ≥16 remained relatively stable across all time points, ranging from 9–11 %. Of those women scoring ≥16 at one interview, 25 % had high scores at two or more other time points. Of all the women taking part in the study 29 % had a score of ≥16 at least once across the assessment period.

In a re-examination of the data, Kaufert and colleagues compared the risk of developing depressive symptoms during the menopausal transition using data from 469 women who had been involved in the initial five interviews conducted as part of the Manitoba Project [42]. The age range of the cohort at baseline was 45–55 with a mean age of 48.4 years. They found that menopausal status did not significantly alter the likelihood of a woman becoming, or staying depressed. Classification into depressed versus non-depressed groups was based on CESD scores, with those scoring higher than 16 at any point of assessment being considered as depressed (or having a history of depression at following time points). For postmenopausal women who had no history of depression the relative odds of developing depression compared to premenopause and perimenopause was 0.87 and for those with a history the relative odds were 0.84 [42]. According to this study menopausal status did not have any impact on the likelihood of developing depression, however women who had undergone surgical menopause were more vulnerable to developing depression. Non-depressed women having undergone hysterectomy had relative odds of 1.7 while those with a history of depression had odds of 0.84.

**Fig. 2:**
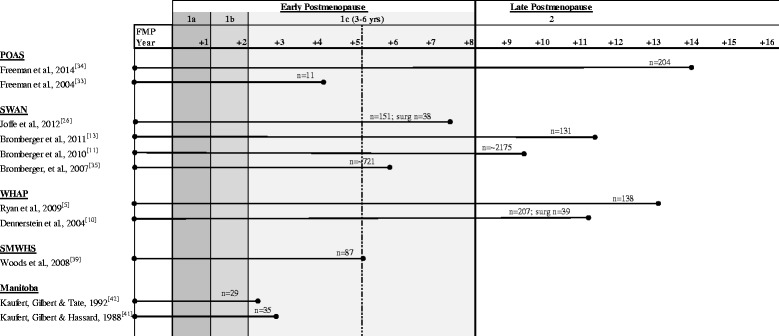
Approximation of range of postmenopause duration by study. Published ranges of the duration of postmenopause for each cohort are included and overlaid on the STRAW + 10 Staging system. *n* = sample size; surg *n* = sample size of surgical menopause; FMP – final menstrual period year and + year since FMP; dashed line – range between 3and 6 years for stage 1c illustrated

A comparison of the maximum duration of the postmenopause reported for each of the subsamples is presented in a standardised format in Fig. 2. An estimation of the maximum years of data available for the postmenopause for each cohort was overlaid on the STRAW +10 staging system for reproductive ageing. The figure highlights the variation in sample sizes used within and between the larger studies. It also shows that the definition of postmenopause used by the studies varies considerably, with some studies describing a period of 2–3 years and others a period of over 15 years [25, 39]. In order to compare findings across studies a more precise indication of years since the final menstrual period should be included.

## Discussion

Standardised depressive symptom measures were used by all of the studies examined in this review. However, not all studies reported on percentage of participants having normal versus mild or moderate symptoms. The SWAN and POAS studies used the CESD full version and the WHAP used the brief version. The Eindhoven study used the EDS. The prevalence scores above the relevant cut offs to determine the presence of mild or moderate depressive symptoms was: 22 % and 25.4 % in WHAP; 24.2 % and 25.7 % in the Eindhoven study. The SWAN study reported higher odds ratio of having depressive symptoms in postmenopause compared to premenopause at 1.57 [30] and 1.79 [10]. For the surgical menopause group prevalence of depressive symptoms was 42 % for WHAP.

In considering clinical diagnoses, SWAN reported 42.4 % of participants met criteria for past or current depressive disorder and 9.8 % for major depressive episode. SWAN also described 31.6 % of the surgical postmenopause group as meeting criteria for past or current depressive disorder. The higher incidence rate is likely impacted by the inclusion of minor depression in the assessment criteria. EPOS reported <1 % Major Depressive Disorder in their postmenopausal sample but used a cut off of 11 or greater on the Edinburgh Depression Scale to determine presence of MDD rather than a clinical assessment.

The prevalence of depressive symptoms was relatively consistent across studies, with a range of 22 % to 25.7 %, (10, 18). Similarly, most studies demonstrated consistency in higher depressive symptom ratings for the surgical postmenopause group. The prevalence of depressive symptoms reported in these longitudinal studies were somewhat higher than those reported by the Women’s Health Initiative Observation Study (WHI-OS), a large prospective study of postmenopausal women carried out in the United States [43]. Baseline characteristics of the 93, 676 multi-ethnic women in this study demonstrated depressive symptom levels at baseline as: 18.1 % for postmenopausal women aged 50–59; 14.8 % for women aged 60–69 and 14.5 % for those aged 70–79 years. Depressive symptoms were assessed using six items from the CESD and two items from the Diagnostic Interview Schedule [43]. The lower reports of depressive symptoms in this cohort may be due to the nature of the enrolment process, with participants representing a sample of convenience rather than being drawn from random population sampling. The prevalence of symptoms reported in this review were also slightly higher than estimates for community dwelling older adults of mixed gender which is thought to be approximately between 12 % and 20 % but varies greatly with prevalence between 0.4 % and 30 % reported in the literature [19, 44]. Studies of older adults or women in late-life cannot be used to generalize to the experiences of postmenopausal women. On average, women in the early postmenopause are aged between 51 and 56–59 years, at which time they enter late postmenopause. The definition of the age at which “late-life” begins is consistently reported as 60-65 years in the literature with “oldest old” age representing those 85 years or greater [19]. Studies that do describe prevalence of depressive symptoms and potential risk factors for women in late-life are greatly impacted by illness associated with old age as well as lifestyle factors unique to this age range such as hospitalisation, loss of loved ones and neurocognitive decline. Women in the postmenopause span mid-life, late life and the oldest old and are not necessarily “older” women as they may have been traditionally viewed.

This review includes data on prevalence reported by longitudinal epidemiological studies exploring mood and the menopausal transition. Any study that included prevalence rates of depressive symptoms or Major Depressive Disorder and reference to the postmenopause specifically were included. However, several of the studies that reported on the postmenopause did so in the context of examining the menopause in general and may not have focused in detail on the postmenopause. For this reason, sample sizes for these populations may be low as they were not the intended focus of the research. Similarly definitions of ‘early’ versus ‘late’ postmenopause may not have been relevant to these studies and as such this distinction was not made in many cases. Studies of ageing have reported prevalence of depressive symptoms by age group, including age ranges relevant to postmenopausal women [44, 45]. However, without definitional criteria relating to postmenopausal status one is unable to draw conclusions about prevalence patterns within the stages of postmenopause. This is particularly true for the first two substages of early postmenopause which may pose a period of vulnerability compared to other stages of the postmenopause [34]. In order to assess this distinct period of reproductive ageing a detailed assessment of the time of the final menstrual period needs to be included as a reference point for the onset of early postmenopause.

Differentiating between the effects of biological ageing and reproductive ageing on mood and depressive symptoms is most accurately captured using longitudinal assessment with repeated measures. Inconsistency in definitions and methodology used between, and even within, these studies highlights the need to apply standardised definitions and assessment tools. The STRAW criteria used in a majority of the studies described here was the gold standard staging system at the time. The updated STRAW + 10 system was developed based on our understanding of documented changes in menstrual, endocrine, and ovarian markers of reproductive aging [9, 16, 17]. Given the importance of the FMP in any examination of the climacteric, it would be beneficial for researchers to include specific information about this event in publications. Not only can it serve as a consistent frame of reference for reproductive age, but also as a means for comparing large data sets and conducting metanalyses regardless of the definition of menopausal status being used.

As our understanding of the postmenopause improves, the application of consistent measures and the use of definitions based on standardised criteria are both crucial to determine the prevalence of depressive symptoms across the postmenopausal transition.

## Conclusions

Prevalence of depressive symptoms in the postmenopause was most consistently reported to be between 22 % to 25 % however, wide variation in study design makes it difficult to accurately estimate the number of women at risk of experiencing depressive symptoms during this time. The term ‘postmenopause’ has been used to group both the early and late postmenopause. Based on recent research the early postmenopause may represent a unique period of vulnerability and must be examined using the more thorough definitional parameters instituted in the new STRAW + 10 criteria. Given the distinct hormonal changes that occur during the substages of the early postmenopause and the higher likelihood of vasomotor symptoms experienced at this time, an assessment of changing risk during this time is essential.

Despite the limitations of currently available data, it is clear that the prevalence of depressive symptoms is high, with roughly a quarter of women experiencing symptoms during the postmenopause. The presence of depressive symptoms is associated with poorer overall functioning, [46] and a decreased quality of life [47]. While depression is the leading cause of neuropsychiatric disability for both sexes, the burden of depression is 50 percent higher for females [48]. Women are living longer and as the population of older women experiencing morbidity grows, it is crucial to identify modifiable risk factors which have the potential to improve mental health and quality of life in this population.
